# Genome-wide identification and analysis of class III peroxidases in *Betula pendula*

**DOI:** 10.1186/s12864-021-07622-1

**Published:** 2021-05-01

**Authors:** Kewei Cai, Huixin Liu, Song Chen, Yi Liu, Xiyang Zhao, Su Chen

**Affiliations:** State Key Laboratory of Tree Genetics and Breeding, Northeast Forestry University, Harbin, 150040 China

**Keywords:** *Betula pendula*, Class III peroxidases, Phylogenetic analysis, Chromosomal location, Expression pattern

## Abstract

**Background:**

Class III peroxidases (POD) proteins are widely present in the plant kingdom that are involved in a broad range of physiological processes including stress responses and lignin polymerization throughout the plant life cycle. At present, *POD* genes have been studied in *Arabidopsis*, rice, poplar, maize and Chinese pear, but there are no reports on the identification and function of *POD* gene family in *Betula pendula.*

**Results:**

We identified 90 nonredundant *POD* genes in *Betula pendula*. (designated *BpPODs*). According to phylogenetic relationships, these *POD* genes were classified into 12 groups. The *BpPODs* are distributed in different numbers on the 14 chromosomes, and some *BpPODs* were located sequentially in tandem on chromosomes. In addition, we analyzed the conserved domains of BpPOD proteins and found that they contain highly conserved motifs. We also investigated their expression patterns in different tissues, the results showed that some *BpPODs* might play an important role in xylem, leaf, root and flower. Furthermore, under low temperature conditions, some *BpPOD*s showed different expression patterns at different times.

**Conclusions:**

The research on the structure and function of the *POD* genes in *Betula pendula* plays a very important role in understanding the growth and development process and the molecular mechanism of stress resistance. These results lay the theoretical foundation for the genetic improvement of *Betula pendula.*

## Background

Peroxidases or peroxide reductases (POD, EC number 1.11.1.x) are a large group of oxidases existing in animals, plants and microorganisms, which catalyzes the oxidation of a particular substrate by hydrogen peroxide [1]. Among them, class III peroxidases are plant specific oxidoreductases, which are extremely widespread presence in the plant kingdom [2]. The Class III peroxidase in plants are also reported as POX [3, 4], GPX [5], Prx [6], ClassIII PRX [7], and POD [8, 9]. Most plant species contain dozens of Class III peroxidases, for example, switchgrass [7] genome contains more than 200 *POD* coding genes, and *Populus* [10], rice and *Arabidopsis* contain 93, 138 and 73 members of POD family, respectively [6, 11].

POD are secreted peroxidase derived from higher plants, participate in a variety of physiological processes in the whole plant life cycle [12]. Recent studies indicate that POD has two most important functions in plants: on the one hand, it is related to the normal morphogenesis of plants and plays a role in the growth and development of plants. On the other hand, it is related to the resistance of plants, including disease resistance, cold resistance, drought resistance, etc., and it is one of the important protective enzymes in plants [13, 14]. Although it is known that POD play a key role in cell growth and response to abiotic stress, the specific function of each member of the family is still elusive. Therefore, it is very important to study the molecular mechanisms of POD in plant development and stress resistance [15].

Gene family is a group of genes derived from the same ancestor, which are composed of two or more copies of a gene through gene doubling or duplication [16]. During the last decade, several molecular biology approaches have been developed to isolate, characterize and study the expression of *POD* gene family in plants [6]. *Betula pendula* is a pioneer boreal tree that can be induced to flower within 1 year [17], it plays an important role in people life [18, 19]. However, so far, there has been no report about the *POD* gene family in *B. pendula*. It has been shown that POD is related to the synthesis of lignin [20] and cork [21, 22], and lignin is considered as an important defense means against invasion and expansion of pathogens [23, 24]. At the same time, a large number of experimental evidences of stress treatment showed that under the stress of drought and low temperature, the expression of POD increased significantly [25, 26].

Since *Betula pendula* is a widespread species and has many applications in the pulp and paper industry, it is necessary to study its development and physiology [27]. Understanding the role of POD family in lignin synthesis and resistance to biotic and abiotic stresses in *B. pendula*, it will contribute to its application in industrial production [28]. Fortunately, with the completion of the whole genome sequencing of *B. pendula* [29, 30], bioinformatics analysis of the *POD* gene family in *B. pendula* at the genome level has become possible.

In the study, we used bioinformatics methods to identify *POD* gene family members in *B. pendula* from the genomic level, and analyzed their protein physical and chemical properties, subcellular localization, evolutionary relationship, conserved motifs and other information [31]. Our study provides important insights for further study of the potential role of *POD* gene family in *B. pendula* growth and development.

## Results

### Identification of *POD* genes

To identify members of *POD* family in *B. pendula*, we used the 73 *POD* genes of *Arabidopsis* to obtain the best hits in the *B. pendula* genome by BLASTP. A total of 90 putative *PODs* were identified in the *B. pendula* genome. We further examined the conserved domains of proteins encoded by these genes using Pfam [32] and SMART [33] databases. The results revealed that all the genes have classical *POD* domain structures, which demonstrate the reliability of the results. The *B. pendula* genome contains more *PODs* than *Arabidopsis* (73) [6], but fewer than *Populus trichocarpa* (93) [34], *Pyrus bretschneideri* (94) [31], and rice (138) [11]. We defined the *BpPODs* as *BpPOD1* to *BpPOD90*. The isoelectric points (PI) ranged from 4.28 to 9.6, and 46 POD proteins were greater than 7.5. In addition, subcellular locations of these *BpPODs* are mainly in the cytoplasm, cell membrane, vacuole, chloroplast and nucleus. The subcellular location, molecular weight (MW) and other information of each *BpPOD* genes was listed in Table 1.

**Table 1:** The 90 POD genes identified in *B. pendula* and their sequence characteristics

Protein Name	Gene ID	Theoretical pI	Molecular weight (Da)	Subcellular localization
BpPOD1	Bpev01.c0000.g0142	7.16	38,520.2	Vacuole
BpPOD2	Bpev01.c0001.g0018	5.76	34,481.79	Cytoplasm
BpPOD3	Bpev01.c0015.g0107	8.52	35,715.62	Cytoplasm
BpPOD4	Bpev01.c0015.g0108	9.06	35,517.27	Cytoplasm
BpPOD5	Bpev01.c0022.g0082	8.05	34,206.63	Cytoplasm
BpPOD6	Bpev01.c0022.g0083	9.11	34,146.78	Cytoplasm
BpPOD7	Bpev01.c0023.g0043	9.32	36,436.39	Cytoplasm
BpPOD8	Bpev01.c0027.g0161	6.43	35,985.96	Cytoplasm
BpPOD9	Bpev01.c0038.g0066	8.86	16,650.95	Cytoplasm
BpPOD10	Bpev01.c0055.g0011	5.7	36,849.2	Cytoplasm
BpPOD11	Bpev01.c0090.g0013	9.15	40,130.67	Cytoplasm
BpPOD12	Bpev01.c0090.g0014	8.72	35,448.63	Cytoplasm
BpPOD13	Bpev01.c0090.g0016	8.9	35,155.75	Cytoplasm
BpPOD14	Bpev01.c0090.g0017	9.03	34,839.38	Cytoplasm
BpPOD15	Bpev01.c0090.g0018	9.21	34,931.7	Cytoplasm
BpPOD16	Bpev01.c0094.g0039	7.57	35,824.75	Cytoplasm
BpPOD17	Bpev01.c0115.g0033	8.28	34,790.11	Cytoplasm
BpPOD18	Bpev01.c0115.g0034	9.21	34,709.95	Cytoplasm
BpPOD19	Bpev01.c0115.g0036	9.57	34,410.61	Cytoplasm
BpPOD20	Bpev01.c0115.g0100	8.13	28,980.85	Cytoplasm
BpPOD21	Bpev01.c0127.g0079	8.51	37,428.88	Cytoplasm
BpPOD22	Bpev01.c0154.g0008	6.98	34,749.39	Cytoplasm
BpPOD23	Bpev01.c0154.g0009	5.97	34,913.58	Cytoplasm
BpPOD24	Bpev01.c0154.g0011	6.17	38,375.7	Cytoplasm
BpPOD25	Bpev01.c0154.g0012	5.71	34,090.42	Cytoplasm
BpPOD26	Bpev01.c0154.g0013	8.56	33,988.06	Cytoplasm
BpPOD27	Bpev01.c0154.g0014	4.92	30,751.05	Cytoplasm
BpPOD28	Bpev01.c0154.g0015	5.79	33,695.64	Cytoplasm
BpPOD29	Bpev01.c0154.g0016	9.09	37,699.91	Cytoplasm
BpPOD30	Bpev01.c0161.g0034	6.95	37,831.82	Cytoplasm
BpPOD31	Bpev01.c0210.g0047	8.01	35,734.78	Cytoplasm
BpPOD32	Bpev01.c0214.g0014	4.7	44,989.41	Cytoplasm
BpPOD33	Bpev01.c0222.g0007	6.09	36,320.35	Cytoplasm
BpPOD34	Bpev01.c0228.g0001	6.29	25,755.32	Chloroplast
BpPOD35	Bpev01.c0253.g0021	6.31	33,855.45	Cytoplasm
BpPOD36	Bpev01.c0253.g0022	4.75	35,040.89	Vacuole
BpPOD37	Bpev01.c0253.g0025	4.28	36,363.54	Vacuole
BpPOD38	Bpev01.c0253.g0026	4.8	36,734.26	Vacuole
BpPOD39	Bpev01.c0292.g0023	6.75	35,088.84	Cytoplasm
BpPOD40	Bpev01.c0335.g0033	5.16	37,438.99	Cytoplasm
BpPOD41	Bpev01.c0395.g0053	4.8	34,822.54	Vacuole
BpPOD42	Bpev01.c0414.g0013	9.23	35,888.05	Cytoplasm
BpPOD43	Bpev01.c0441.g0005	7.52	35,297.73	Cytoplasm
BpPOD44	Bpev01.c0443.g0013	6.51	37,358.81	Cytoplasm
BpPOD45	Bpev01.c0483.g0021	5.6	36,858.73	Cytoplasm
BpPOD46	Bpev01.c0518.g0009	6.34	35,401.21	Cytoplasm
BpPOD47	Bpev01.c0518.g0010	6.22	35,012.98	Cytoplasm
BpPOD48	Bpev01.c0566.g0037	4.74	35,256.87	Cytoplasm
BpPOD49	Bpev01.c0577.g0019	8.86	33,926.77	Cytoplasm
BpPOD50	Bpev01.c0605.g0023	5.58	37,438.76	Cytoplasm
BpPOD51	Bpev01.c0605.g0024	5.92	40,256.6	Cytoplasm
BpPOD52	Bpev01.c0672.g0007	5.31	35,421.93	Cytoplasm
BpPOD53	Bpev01.c0702.g0001	8.28	41,401.42	Cytoplasm
BpPOD54	Bpev01.c0753.g0001	5.97	23,067.41	Cytoplasm
BpPOD55	Bpev01.c0811.g0007	8.7	9122.73	Cell membrane
BpPOD56	Bpev01.c0834.g0015	7.95	37,636.09	Cytoplasm
BpPOD57	Bpev01.c0848.g0029	8.46	36,912.4	Cytoplasm
BpPOD58	Bpev01.c0932.g0013	4.69	34,485.93	Cytoplasm
BpPOD59	Bpev01.c0944.g0009	9.6	35,965.28	Cytoplasm
BpPOD60	Bpev01.c0990.g0011	8.86	34,411.46	Cytoplasm
BpPOD61	Bpev01.c0991.g0009	9.37	16,644.16	Cytoplasm
BpPOD62	Bpev01.c1029.g0016	4.71	38,697.61	Cytoplasm
BpPOD63	Bpev01.c1029.g0017	5.2	38,867.05	Cytoplasm
BpPOD64	Bpev01.c1078.g0006	5.67	17,097.87	Cell membrane
BpPOD65	Bpev01.c1163.g0010	8.1	36,508.06	Cytoplasm
BpPOD66	Bpev01.c1189.g0010	6.93	35,457.58	Cytoplasm
BpPOD67	Bpev01.c1189.g0011	5.94	28,953.96	Cytoplasm
BpPOD68	Bpev01.c1230.g0004	6.41	57,999.02	Cytoplasm
BpPOD69	Bpev01.c1230.g0005	8.95	37,658.16	Cytoplasm
BpPOD70	Bpev01.c1519.g0002	6.99	35,815.14	Cytoplasm
BpPOD71	Bpev01.c1529.g0006	8.89	38,531.35	Cytoplasm
BpPOD72	Bpev01.c1719.g0005	8.42	33,743.46	Cytoplasm
BpPOD73	Bpev01.c1776.g0001	8.38	33,425.87	Cytoplasm
BpPOD74	Bpev01.c1776.g0002	6.44	28,814.32	Cytoplasm
BpPOD75	Bpev01.c1889.g0001	8.46	32,601.89	Cytoplasm
BpPOD76	Bpev01.c1889.g0002	8.75	43,372.13	Cytoplasm
BpPOD77	Bpev01.c1889.g0003	8.05	33,592.04	Cytoplasm
BpPOD78	Bpev01.c1922.g0001	8.42	34,940.65	Cytoplasm
BpPOD79	Bpev01.c1922.g0002	9.41	32,305.51	Cytoplasm
BpPOD80	Bpev01.c2035.g0001	5.3	20,474.83	Chloroplast
BpPOD81	Bpev01.c2059.g0007	7.56	34,908.57	Cytoplasm
BpPOD82	Bpev01.c2165.g0002	6.38	34,883.46	Cytoplasm
BpPOD83	Bpev01.c2185.g0001	5.01	14,822	Nucleus
BpPOD84	Bpev01.c2220.g0001	9.04	29,887.06	Cytoplasm
BpPOD85	Bpev01.c2322.g0001	9.35	35,748.32	Cytoplasm
BpPOD86	Bpev01.c3133.g0001	6.89	9127.53	Nucleus
BpPOD87	Bpev01.c3133.g0002	7.89	61,365.35	Cytoplasm
BpPOD88	Bpev01.c3139.g0001	8.54	34,756.42	Cytoplasm
BpPOD89	Bpev01.c3210.g0001	8.53	33,682.38	Cytoplasm
BpPOD90	Bpev01.c3916.g0001	4.74	38,628.55	Cytoplasm

### Phylogenetic analyses of *POD* gene family in *B. pendula*

**Fig. 1:**
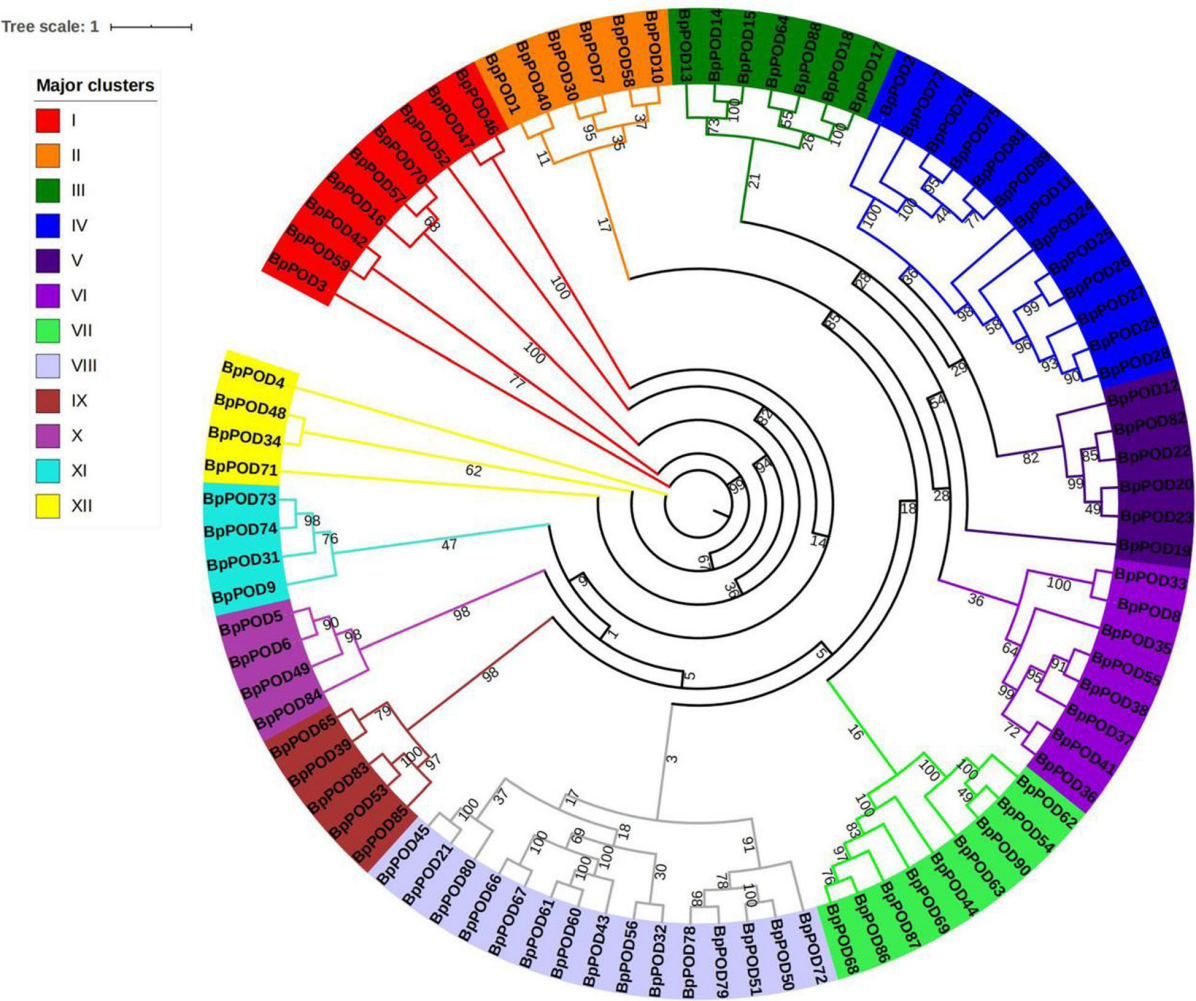
Phylogenetic analysis of *POD* family genes from *B. pendula*. According to phylogenetic relationships, 90 *BpPOD* genes were classified into 12 subgroups (subgroups I to XII). The phylogenetic tree was constructed using MEGA 7.0 with the maximum likelihood (ML) method

To investigate the evolutionary relationships, we performed multiple sequence alignment of *POD* family genes in *B. pendula* and *Arabidopsis*, and constructed the phylogenetic tree by MEGA 7.0 software (Fig. 1). The BpPOD proteins were classified into 12 groups with high bootstrap probabilities, designated group I to group XII. The *POD* genes of each subgroup is unevenly distributed, with the number of members varies from 4 to 15. Subgroup VIII contains the most members (15), subgroup X, XI, XII contains the least number of members, with only 4 members.

### Gene structures

**Fig. 2:**
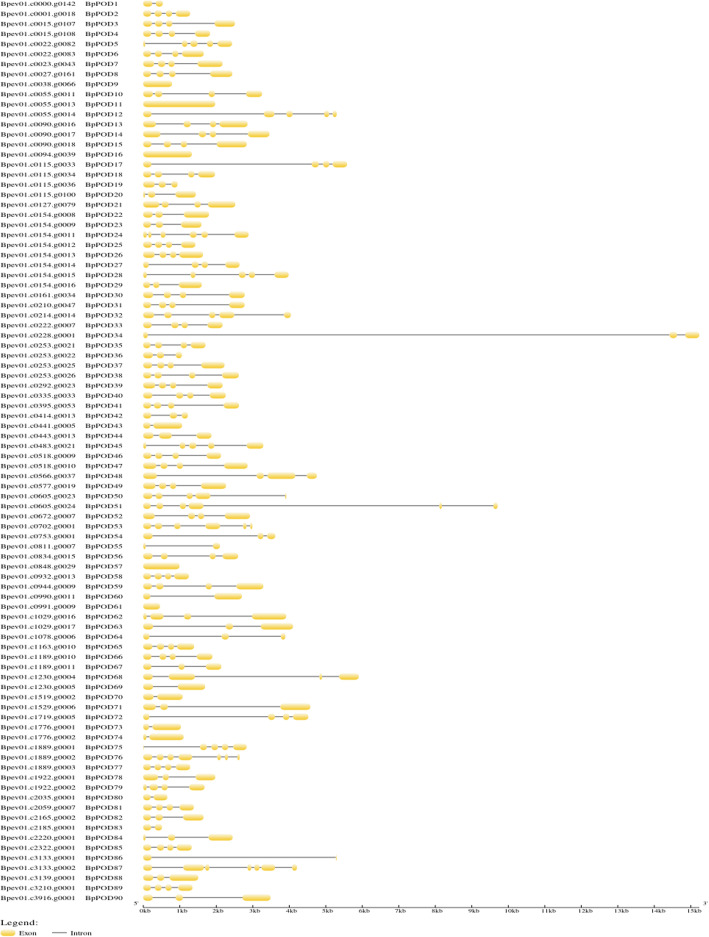
Gene structure analyses of *BpPOD* genes. The exons and introns are indicated by yellow cylinder bars and black lines, respectively. The scale at the bottom of the figure is in kilobases. Gene structure was visualized by Gene Structure Display Server (GSDS)

To understand the structural diversity of the *POD* genes, exon-intron analysis was performed in *BpPODs* (Fig. 2)*.* The result reveals several variations, in terms of the number of introns, *BpPODs* contains one to six introns, and some members contain three introns. Noteworthy, there were no introns in five *BpPODs* (*BpPOD9, BpPOD11, BpPOD16, BpPOD57 and BpPOD61*). In addition, *BpPOD76 and BpPOD87* have the most introns (6), followed by *BpPOD24* and *BpPOD51* (5). Moreover, we found that the genes of the same group are similar in gene structure. For example, *BpPOD20, BpPOD22 and BpPOD82* have three exons and two intron, both of which belong to Group V; *BpPOD73 and BpPOD74* have two exons and one intron, both of which belong to Group XI [35].

### Analysis of conserved amino acid motifs

**Fig. 3:**
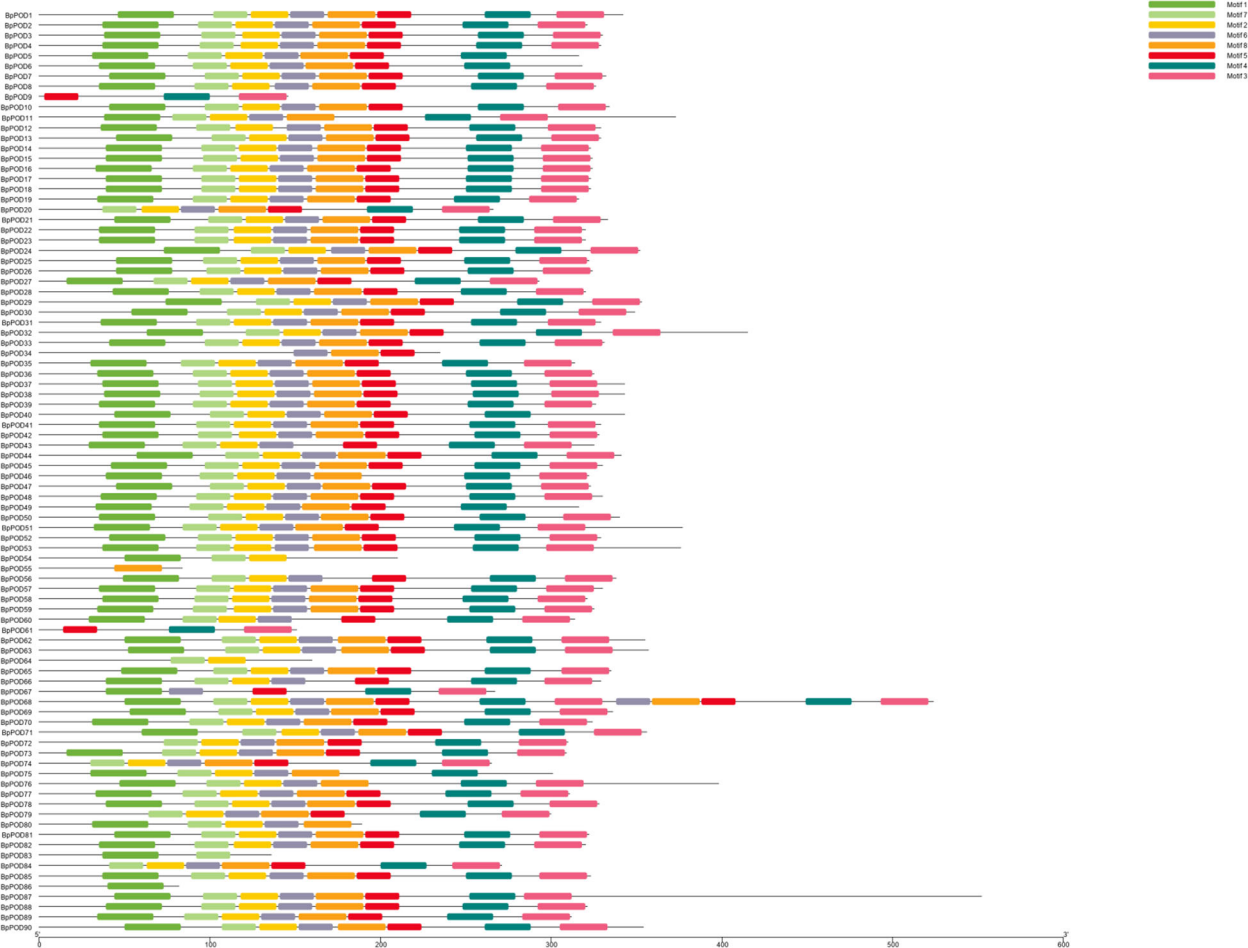
The conserved motifs of 90 BpPOD proteins. Conserved motifs are represented by different colored boxes while nonconserved sequences are shown by gray lines. The conserved motif figure of BpPOD proteins was visualized by TBtools software

To understand the functional regions of BpPODs, conserved amino acid motifs analyses of BpPOD proteins were performed. A total of eight conserved amino acid motifs were identified in the BpPOD proteins (Fig. 3). All BpPOD proteins contain at least one conserved amino acid motif. For example, BpPOD55 only contains motif 8, BpPOD83 contains motif 1 and 7, while BpPOD10 proteins contain all the eight conserved amino acid motifs.

The conserved motifs of POD proteins clustered in the same group are similar in composition, indicating that these members have close evolutionary relationships [36]. In addition, most members of BpPOD proteins contain motif 1, motif 2, motif 3, motif 4 and other conserved motifs, these motifs might play an important role in BpPOD proteins.

### Chromosomal location and evolution analysis of *BpPODs*

**Fig. 4:**
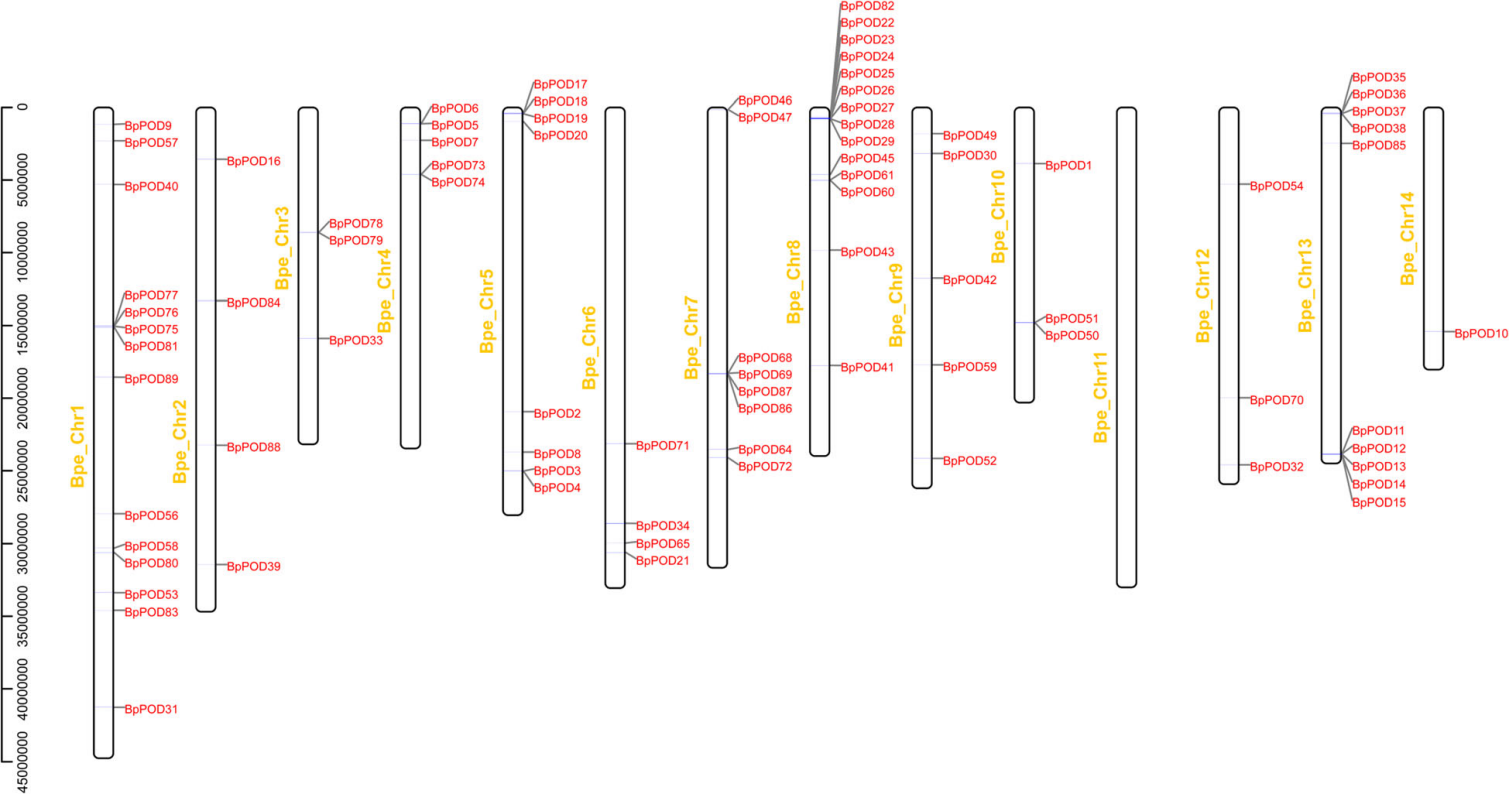
Chromosomal locations of 90 *POD* genes on 14 *B. pendula* chromosomes. The number of chromosomes (chr01-chr14) is marked in yellow, and each *POD* gene is marked in red. The gene names on the right side of each chromosome correspond to the approximate locations of each *POD* gene. The chromosome location figure of *BpPODs* was constructed by TBtools software

Based on the genomic information of *B. pendula*, we analyzed the chromosomal distribution of 90 *BpPODs*. Chromosome localization analysis showed that the 90 *BpPODs* were unevenly distributed on 14 chromosomes (Fig. 4). Chromosome 1 and 8 contains the most *BpPOD*s (14), followed by chromosome 13 (10). There are eight *BpPODs* on chromosome 5 and chromosome 7, and only one *BpPODs* on chromosome 14. Noteworthy, there is no *POD* gene distribution on chromosome 11. We also found that the relatively high density of *BpPODs* on chromosome 13 and chromosome 8.

**Fig. 5:**
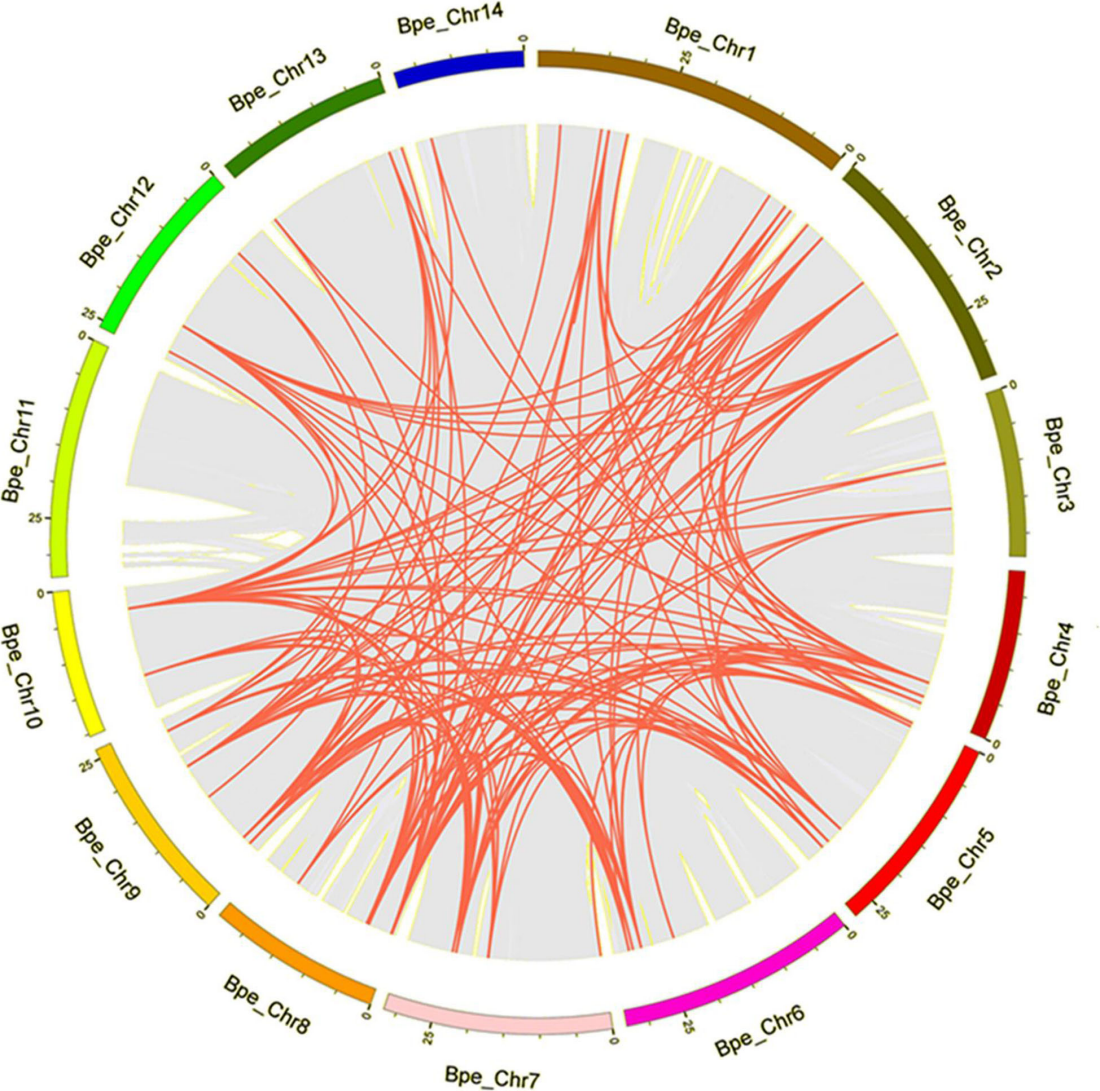
*BpPODs* genomic distribution and collinear relationships. A total of 90 *BpPODs* were disproportionately mapped on the *Betula pendula* linkage groups using TBtools software. Red lines represent all homologous blocks in *Betula pendula* genome

Gene duplication, including segmental and tandem duplication, is considered to be one of the primary driving forces in the evolution of genomes [37, 38]. In this study, among the 90 *BpPODs* identified, a large number of *BpPODs* have the same duplicated regions (Fig. 5). In general, gene tandem duplication is one of the basic reasons for the formation of gene clusters [39]. In this study, we found that some *BpPODs* were adjacent to each other (Fig. 4). For instance, *BpPOD17–20* on chromosome 5, *BpPOD22–29* on chromosome 8, and *BpPOD11–15* on chromosome 13 were tandemly linked together, implying that tandem duplication relationships may exist between these *BpPODs* [40]. The result indicated that tandem duplications play main contributors in the expansion of the *BpPOD* gene family. The result was consistent with *Populus trichocarpa POD* gene family, tandem duplications also contributed significantly to the expansion of *POD* gene family in *Populus trichocarpa* [34]. However, in previous studies, many species also have produced some different results. For example, in the report on the *POD* gene family of pear, it was found that segmental duplication was the main reason for the extension of the *POD* family [31]. In the maize, segmental and tandem duplication affect the extension of maize *POD* gene family [36]. These results indicate that there are significant differences in the *POD* genes expansion pattern in *B. pendula*, maize and Chinese pear, which suggested that *POD* gene family have different expansion patterns among different species.

Considering the selection pressures of the *BpPOD* duplicated genes, Ka, Ks, and Ka/Ks ratios were calculated for the 23gene pairs (Table 2). In the process of evolution, Ka/Ks > 1 represents positive selection, Ka/Ks = 1 represents neutral selection and Ka/Ks < 1 represents negative selection [35]. Ka/Ks analysis showed that the Ka/Ks value of most *BpPOD* gene pairs were less than 1, indicating that these genes underwent negative selection and were relatively conservative in evolution, with relatively stable structure and consistent function.

**Table 2:** The Ka, Ks, and Ka/Ks values for the 23 gene pairs

Paralogous pairs	Ka	Ks	Ka/Ks	Negative selection
BpPOD17-BpPOD18	0.069471117	0.268358877	0.258873928	Yes
BpPOD18-BpPOD20	0.362725757	2.627815477	0.138033192	Yes
BpPOD24-BpPOD25	0.263859296	0.521302694	0.506153717	Yes
BpPOD24-BpPOD26	0.238387956	0.604227441	0.394533482	Yes
BpPOD24-BpPOD27	0.180902743	0.42607505	0.424579525	Yes
BpPOD24-BpPOD28	0.215885332	0.570985748	0.37809233	Yes
BpPOD25-BpPOD26	0.069221668	0.226863803	0.305124339	Yes
BpPOD25-BpPOD27	0.103222209	0.340981758	0.302720619	Yes
BpPOD25-BpPOD28	0.178403774	0.484390738	0.368305503	Yes
BpPOD26-BpPOD27	0.081063342	0.4345943	0.186526474	Yes
BpPOD26-BpPOD28	0.137670156	0.465437767	0.295786387	Yes
BpPOD27-BpPOD28	0.052823689	0.216841185	0.243605425	Yes
BpPOD11-BpPOD12	0.493471639	1.939854071	0.254385959	Yes
BpPOD11-BpPOD14	0.380143832	3.143972492	0.120911946	Yes
BpPOD12-BpPOD14	0.375233519	3.645948628	0.102917939	Yes
BpPOD12-BpPOD15	0.386625798	3.001334218	0.128817976	Yes
BpPOD13-BpPOD14	0.193518905	0.467304328	0.414117511	Yes
BpPOD13-BpPOD15	0.213861204	0.549200628	0.389404514	Yes
BpPOD14-BpPOD15	0.098858095	0.305317793	0.323787532	Yes
BpPOD22-BpPOD23	0.042284565	0.18631819	0.226948131	Yes
BpPOD5-BpPOD6	0.128227162	0.183810398	0.697605596	Yes
BpPOD5-BpPOD7	0.615552629	2.238568932	0.274975954	Yes
BpPOD6-BpPOD7	0.622751118	2.444971397	0.254706914	Yes

**Fig. 6:**
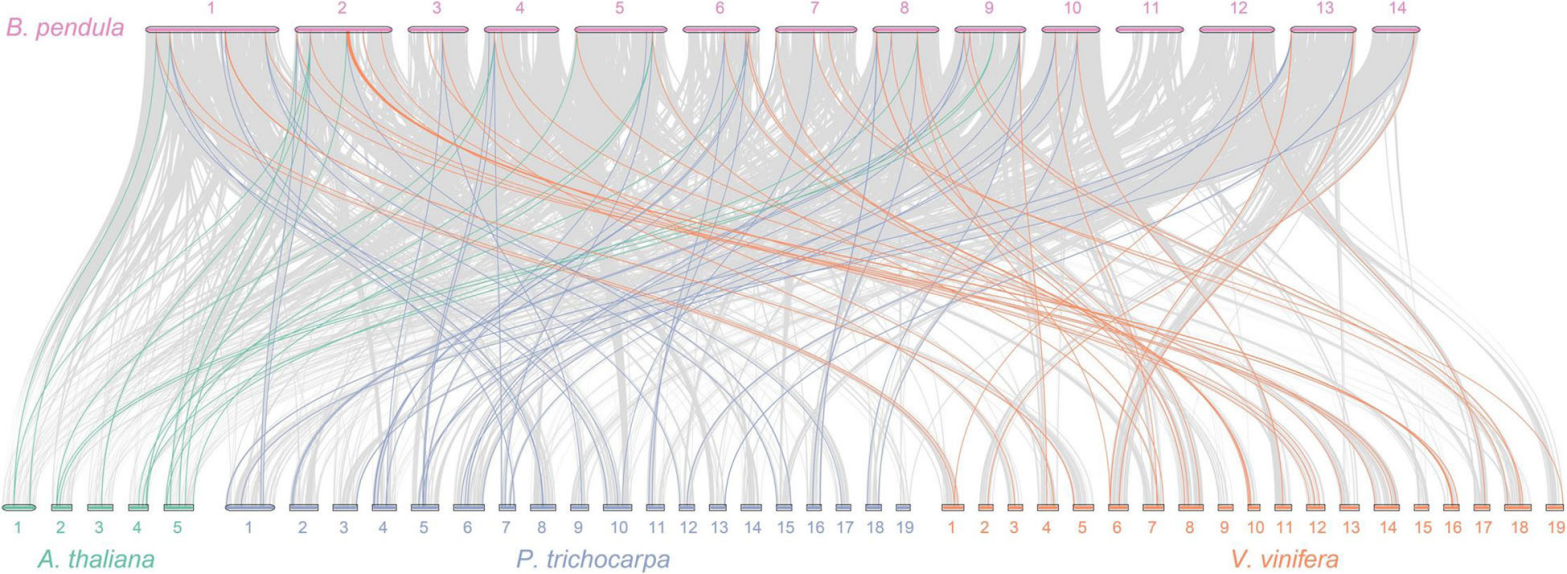
Synteny map of *POD* family genes between *B. pendula* and three representative species. Gray lines indicate all syntenic blocks among birch linkage groups or between birch and the other species. In three species, collinear pairs of *BpPODs* are connected by green, blue and orange lines. Syntenic maps of birch associated with three representative species were visualized by MCScanX

To analyze the evolution of *BpPODs* family, we created the comparative syntenic diagram of the birch and three representative species (Fig. 6; Tables 3, 4 and 5). The results showed that the number of orthologous pairs between *B. pendula* and *Arabidopsis thaliana*, *Populus trichocarpa* and *Vitis vinifera* were 17, 49 and 43, respectively. In these gene pairs, some *BpPOD* genes (*BpPOD3*, *BpPOD7*, *BpPOD16*, *BpPOD21*, *BpPOD40*, *BpPOD4*, *BpPOD48*, *BpPOD52*, *BpPOD57* and *BpPOD84*) were indicated to have collinear relationships with three species. Interestingly, two or more *POD* genes from *Arabidopsis thaliana*, *Populus trichocarpa* and *Vitis vinifera* matched one birch *POD* gene, these genes may play a more important role than other genes in *BpPOD* family. For example, AT1G24110.1.TAIR10, AT3G28200.1.TAIR10 and AT5G40150.1.TAIR10 are orthologous to *BpPOD16*, Potri.006G107000.1.v4.1 and Potri.016G132800.1.v4.1 are orthologous to *BpPOD22*, VIT_206s0004g01180.1 and VIT_208s0007g06650.1 are orthologous to *BpPOD41* (Tables 3, 4 and 5).

**Table 3:** Syntenic relationships of POD genes between *Betula pendula* and *Arabidopsis thaliana*

BpPOD gene name	BpPOD gene ID	AtPOD gene ID
BpPOD3	Bpev01.c0015.g0107.mRNA1	AT4G37520.1.TAIR10
BpPOD3	Bpev01.c0015.g0107.mRNA1	AT5G67400.1.TAIR10
BpPOD7	Bpev01.c0023.g0043.mRNA1	AT2G18150.1.TAIR10
BpPOD7	Bpev01.c0023.g0043.mRNA1	AT4G36430.1.TAIR10
BpPOD16	Bpev01.c0094.g0039.mRNA1	AT1G24110.1.TAIR10
BpPOD16	Bpev01.c0094.g0039.mRNA1	AT3G28200.1.TAIR10
BpPOD16	Bpev01.c0094.g0039.mRNA1	AT5G40150.1.TAIR10
BpPOD17	Bpev01.c0115.g0033.mRNA1	AT5G05340.1.TAIR10
BpPOD21	Bpev01.c0127.g0079.mRNA1	AT4G21960.1.TAIR10
BpPOD40	Bpev01.c0335.g0033.mRNA1	AT1G68850.1.TAIR10
BpPOD41	Bpev01.c0395.g0053.mRNA1	AT5G06730.1.TAIR10
BpPOD42	Bpev01.c0414.g0013.mRNA1	AT2G18980.1.TAIR10
BpPOD42	Bpev01.c0414.g0013.mRNA1	AT4G30170.1.TAIR10
BpPOD48	Bpev01.c0566.g0037.mRNA1	AT5G14130.1.TAIR10
BpPOD52	Bpev01.c0672.g0007.mRNA1	AT2G24800.1.TAIR10
BpPOD57	Bpev01.c0848.g0029.mRNA1	AT1G24110.1.TAIR10
BpPOD84	Bpev01.c2220.g0001.mRNA1	AT5G51890.1.TAIR10

**Table 4:** Syntenic relationships of POD genes between *Betula pendula* and *Populus trichocarpa*

BpPOD gene name	BpPOD gene ID	PtPOD gene ID
BpPOD1	Bpev01.c0000.g0142.mRNA1	Potri.005G072800.1.v4.1
BpPOD1	Bpev01.c0000.g0142.mRNA1	Potri.007G096200.1.v4.1
BpPOD3	Bpev01.c0015.g0107.mRNA1	Potri.007G053400.1.v4.1
BpPOD5	Bpev01.c0022.g0082.mRNA1	Potri.005G108900.1.v4.1
BpPOD7	Bpev01.c0023.g0043.mRNA1	Potri.005G118700.1.v4.1
BpPOD7	Bpev01.c0023.g0043.mRNA1	Potri.007G019300.1.v4.1
BpPOD8	Bpev01.c0027.g0161.mRNA1	Potri.005G135300.1.v4.1
BpPOD10	Bpev01.c0055.g0011.mRNA1	Potri.001G145800.1.v4.1
BpPOD11	Bpev01.c0090.g0013.mRNA1	Potri.013G154400.1.v4.1
BpPOD12	Bpev01.c0090.g0014.mRNA1	Potri.013G156800.1.v4.1
BpPOD14	Bpev01.c0090.g0017.mRNA2	Potri.013G156400.2.v4.1
BpPOD16	Bpev01.c0094.g0039.mRNA1	Potri.001G351000.3.v4.1
BpPOD21	Bpev01.c0127.g0079.mRNA1	Potri.004G015300.2.v4.1
BpPOD22	Bpev01.c0154.g0008.mRNA1	Potri.006G107000.1.v4.1
BpPOD22	Bpev01.c0154.g0008.mRNA1	Potri.016G132800.1.v4.1
BpPOD28	Bpev01.c0154.g0015.mRNA1	Potri.016G132700.1.v4.1
BpPOD30	Bpev01.c0161.g0034.mRNA1	Potri.002G031200.1.v4.1
BpPOD31	Bpev01.c0210.g0047.mRNA1	Potri.012G006800.3.v4.1
BpPOD31	Bpev01.c0210.g0047.mRNA1	Potri.015G003500.1.v4.1
BpPOD33	Bpev01.c0222.g0007.mRNA1	Potri.004G144600.1.v4.1
BpPOD33	Bpev01.c0222.g0007.mRNA1	Potri.009G106400.2.v4.1
BpPOD35	Bpev01.c0253.g0021.mRNA1	Potri.003G214500.1.v4.1
BpPOD36	Bpev01.c0253.g0022.mRNA1	Potri.001G011500.1.v4.1
BpPOD38	Bpev01.c0253.g0026.mRNA1	Potri.001G011000.1.v4.1
BpPOD38	Bpev01.c0253.g0026.mRNA1	Potri.001G012901.1.v4.1
BpPOD38	Bpev01.c0253.g0026.mRNA1	Potri.003G214800.1.v4.1
BpPOD40	Bpev01.c0335.g0033.mRNA1	Potri.008G110600.2.v4.1
BpPOD40	Bpev01.c0335.g0033.mRNA1	Potri.010G134500.1.v4.1
BpPOD41	Bpev01.c0395.g0053.mRNA1	Potri.016G058200.1.v4.1
BpPOD45	Bpev01.c0483.g0021.mRNA1	Potri.006G129900.1.v4.1
BpPOD47	Bpev01.c0518.g0010.mRNA1	Potri.004G134800.1.v4.1
BpPOD48	Bpev01.c0566.g0037.mRNA1	Potri.001G329200.1.v4.1
BpPOD48	Bpev01.c0566.g0037.mRNA1	Potri.017G064100.1.v4.1
BpPOD49	Bpev01.c0577.g0019.mRNA1	Potri.002G018000.1.v4.1
BpPOD49	Bpev01.c0577.g0019.mRNA1	Potri.005G108900.1.v4.1
BpPOD51	Bpev01.c0605.g0024.mRNA1	Potri.006G069600.1.v4.1
BpPOD51	Bpev01.c0605.g0024.mRNA1	Potri.018G131600.1.v4.1
BpPOD52	Bpev01.c0672.g0007.mRNA1	Potri.006G267400.1.v4.1
BpPOD52	Bpev01.c0672.g0007.mRNA1	Potri.018G015500.1.v4.1
BpPOD56	Bpev01.c0834.g0015.mRNA1	Potri.007G132800.1.v4.1
BpPOD57	Bpev01.c0848.g0029.mRNA1	Potri.010G036100.1.v4.1
BpPOD58	Bpev01.c0932.g0013.mRNA1	Potri.008G103200.1.v4.1
BpPOD65	Bpev01.c1163.g0010.mRNA1	Potri.004G023200.1.v4.1
BpPOD65	Bpev01.c1163.g0010.mRNA1	Potri.011G027300.1.v4.1
BpPOD68	Bpev01.c1230.g0004.mRNA1	Potri.010G175100.1.v4.1
BpPOD70	Bpev01.c1519.g0002.mRNA1	Potri.012G076500.1.v4.1
BpPOD71	Bpev01.c1529.g0006.mRNA1	Potri.004G052100.1.v4.1
BpPOD71	Bpev01.c1529.g0006.mRNA1	Potri.011G062300.1.v4.1
BpPOD84	Bpev01.c2220.g0001.mRNA1	Potri.015G138300.1.v4.1

**Table 5:** Syntenic relationships of POD genes between *Betula pendula* and *Vitis vinifera*

BpPOD gene name	BpPOD gene ID	VvPOD gene ID
BpPOD1	Bpev01.c0000.g0142.mRNA1	VIT_207s0191g00050.1.v2.1
BpPOD3	Bpev01.c0015.g0107.mRNA1	VIT_207s0129g00360.1.v2.1
BpPOD7	Bpev01.c0023.g0043.mRNA1	VIT_204s0023g02570.1.v2.1
BpPOD10	Bpev01.c0055.g0011.mRNA1	VIT_202s0012g00540.1.v2.1
BpPOD11	Bpev01.c0090.g0013.mRNA1	VIT_206s0004g07740.1.v2.1
BpPOD12	Bpev01.c0090.g0014.mRNA1	VIT_206s0004g07750.1.v2.1
BpPOD14	Bpev01.c0090.g0017.mRNA2	VIT_206s0004g07770.1.v2.1
BpPOD16	Bpev01.c0094.g0039.mRNA1	VIT_214s0066g01850.1.v2.1
BpPOD17	Bpev01.c0115.g0033.mRNA1	VIT_213s0067g02360.1.v2.1
BpPOD21	Bpev01.c0127.g0079.mRNA1	VIT_210s0116g01780.1.v2.1
BpPOD22	Bpev01.c0154.g0008.mRNA1	VIT_208s0058g00990.1.v2.1
BpPOD28	Bpev01.c0154.g0015.mRNA1	VIT_208s0058g00970.1.v2.1
BpPOD30	Bpev01.c0161.g0034.mRNA1	VIT_218s0001g13110.1.v2.1
BpPOD31	Bpev01.c0210.g0047.mRNA1	VIT_216s0098g00820.1.v2.1
BpPOD33	Bpev01.c0222.g0007.mRNA1	VIT_203s0063g01040.1.v2.1
BpPOD35	Bpev01.c0253.g0021.mRNA1	VIT_206s0004g01240.2.v2.1
BpPOD36	Bpev01.c0253.g0022.mRNA1	VIT_206s0004g01190.1.v2.1
BpPOD38	Bpev01.c0253.g0026.mRNA1	VIT_206s0004g01180.1.v2.1
BpPOD39	Bpev01.c0292.g0023.mRNA1	VIT_212s0059g02420.1.v2.1
BpPOD40	Bpev01.c0335.g0033.mRNA1	VIT_201s0010g01090.1.v2.1
BpPOD41	Bpev01.c0395.g0053.mRNA1	VIT_206s0004g01180.1.v2.1
BpPOD41	Bpev01.c0395.g0053.mRNA1	VIT_208s0007g06650.1.v2.1
BpPOD45	Bpev01.c0483.g0021.mRNA1	VIT_208s0040g02200.1.v2.1
BpPOD47	Bpev01.c0518.g0010.mRNA1	VIT_207s0130g00220.1.v2.1
BpPOD48	Bpev01.c0566.g0037.mRNA1	VIT_214s0068g01920.1.v2.1
BpPOD49	Bpev01.c0577.g0019.mRNA1	VIT_218s0001g01140.1.v2.1
BpPOD51	Bpev01.c0605.g0024.mRNA1	VIT_211s0016g05280.1.v2.1
BpPOD52	Bpev01.c0672.g0007.mRNA1	VIT_204s0008g07040.1.v2.1
BpPOD57	Bpev01.c0848.g0029.mRNA1	VIT_201s0026g00830.1.v2.1
BpPOD58	Bpev01.c0932.g0013.mRNA1	VIT_205s0077g00720.1.v2.1
BpPOD65	Bpev01.c1163.g0010.mRNA1	VIT_210s0116g00340.1.v2.1
BpPOD68	Bpev01.c1230.g0004.mRNA1	VIT_219s0085g01040.1.v2.1
BpPOD70	Bpev01.c1519.g0002.mRNA1	VIT_201s0026g00830.1.v2.1
BpPOD70	Bpev01.c1519.g0002.mRNA1	VIT_217s0000g07750.1.v2.1
BpPOD71	Bpev01.c1529.g0006.mRNA1	VIT_210s0003g00650.1.v2.1
BpPOD72	Bpev01.c1719.g0005.mRNA1	VIT_218s0001g15390.1.v2.1
BpPOD74	Bpev01.c1776.g0002.mRNA1	VIT_214s0060g00510.1.v2.1
BpPOD78	Bpev01.c1922.g0001.mRNA1	VIT_212s0055g00980.1.v2.1
BpPOD80	Bpev01.c2035.g0001.mRNA1	VIT_205s0077g00880.1.v2.1
BpPOD84	Bpev01.c2220.g0001.mRNA1	VIT_216s0100g00740.1.v2.1
BpPOD84	Bpev01.c2220.g0001.mRNA1	VIT_216s0022g02470.1.v2.1
BpPOD84	Bpev01.c2220.g0001.mRNA1	VIT_216s0100g00090.1.v2.1
BpPOD88	Bpev01.c3139.g0001.mRNA1	VIT_212s0028g01840.1.v2.1

### Tissue-specific expression of *BpPODs*

**Fig. 7:**
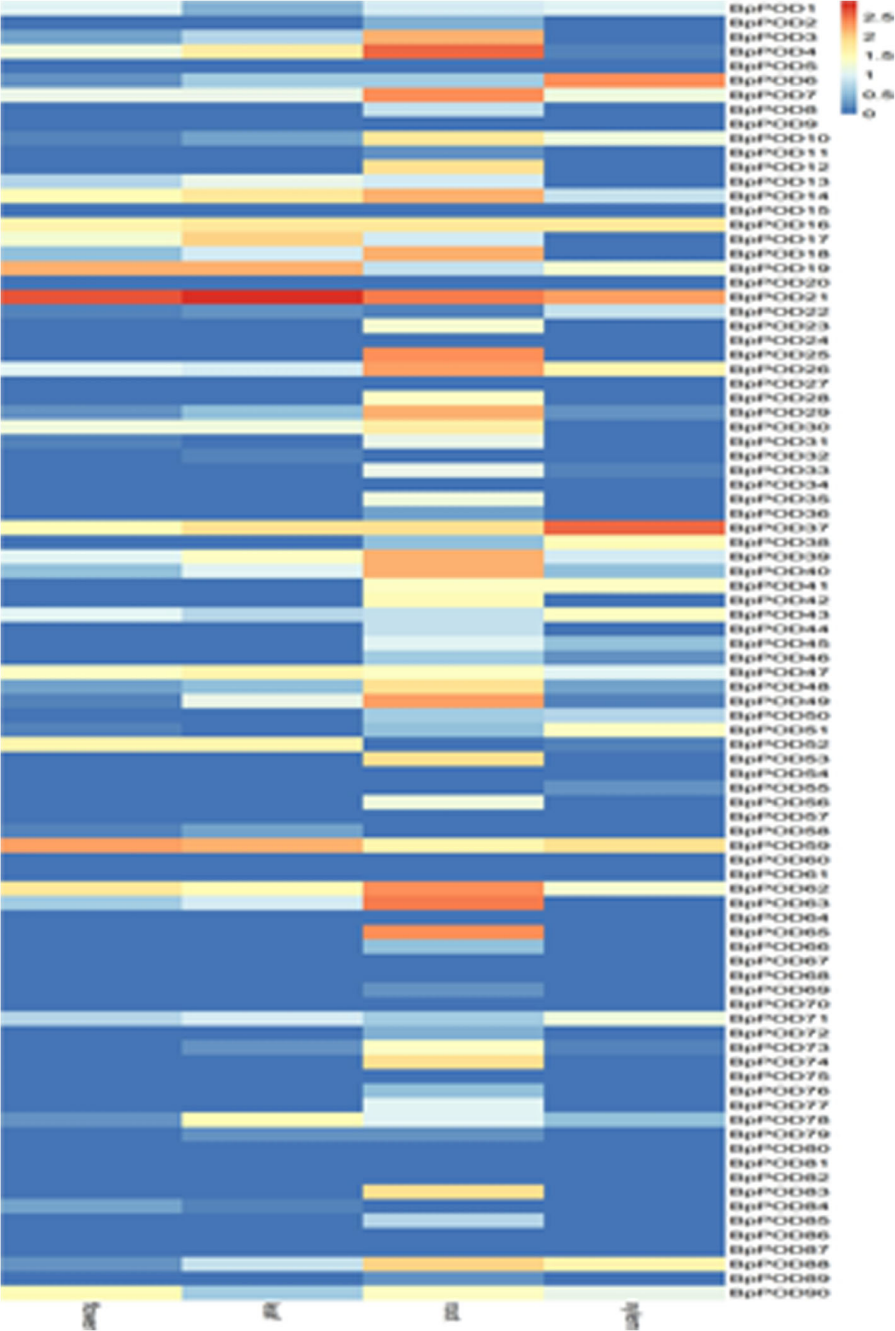
Expression profiles of *BpPOD* genes across different tissues. Different tissues are exhibited below each column. The *BpPOD* genes were listed at the right of the expression array, and the colour box from blue (0) to orange (2.5) indicate an increased expression level is shown at the right of the figure. Color scale represent the normalized value of the expression. For the sake of unified comparison, the normalized value of the expression was log10 transformed

To explore the functions of *POD* genes in *Betula platyphylla* × *Betula pendula*, the expression profiles in different tissues (including root, xylem, young leaf and flower) were investigated with available experimental data. Of the 90 *BpPODs*, 69 genes were expressed in one or more birch tissues, while 21 *BpPOD* genes were not expressed in different tissues (Relative expression value > 0 as basal expression) [41]. As shown in Fig. 7, most *BpPODs* were expressed preferentially in different tissues. For example, *BpPOD6*, *BpPOD21* and *BpPOD37* were highly expressed in xylem. Several *BpPODs* were expressed in root during development, such as *BpPOD62, BpPOD63* and *BpPOD65*. *BpPOD78* and *BpPOD19* showed higher expression levels in young leaf and flower, respectively. The expression level of *BpPOD6* was high in xylem and low in root, leaf and flower. In contrast, *BpPOD67, BpPOD68, BpPOD80* and *BpPOD81* had no expression in any of the investigated tissues. *BpPOD21, BpPOD59* and *BpPOD62* were highly expressed in developing xylem, root, leaf and flower. In conclusion, the expression changes of *BpPODs* in these tissues indicated that *POD* genes played an important role in the growth and development of *B. pendula*.

### Responses of *BpPODs* expression to cold treatment

**Fig. 8:**
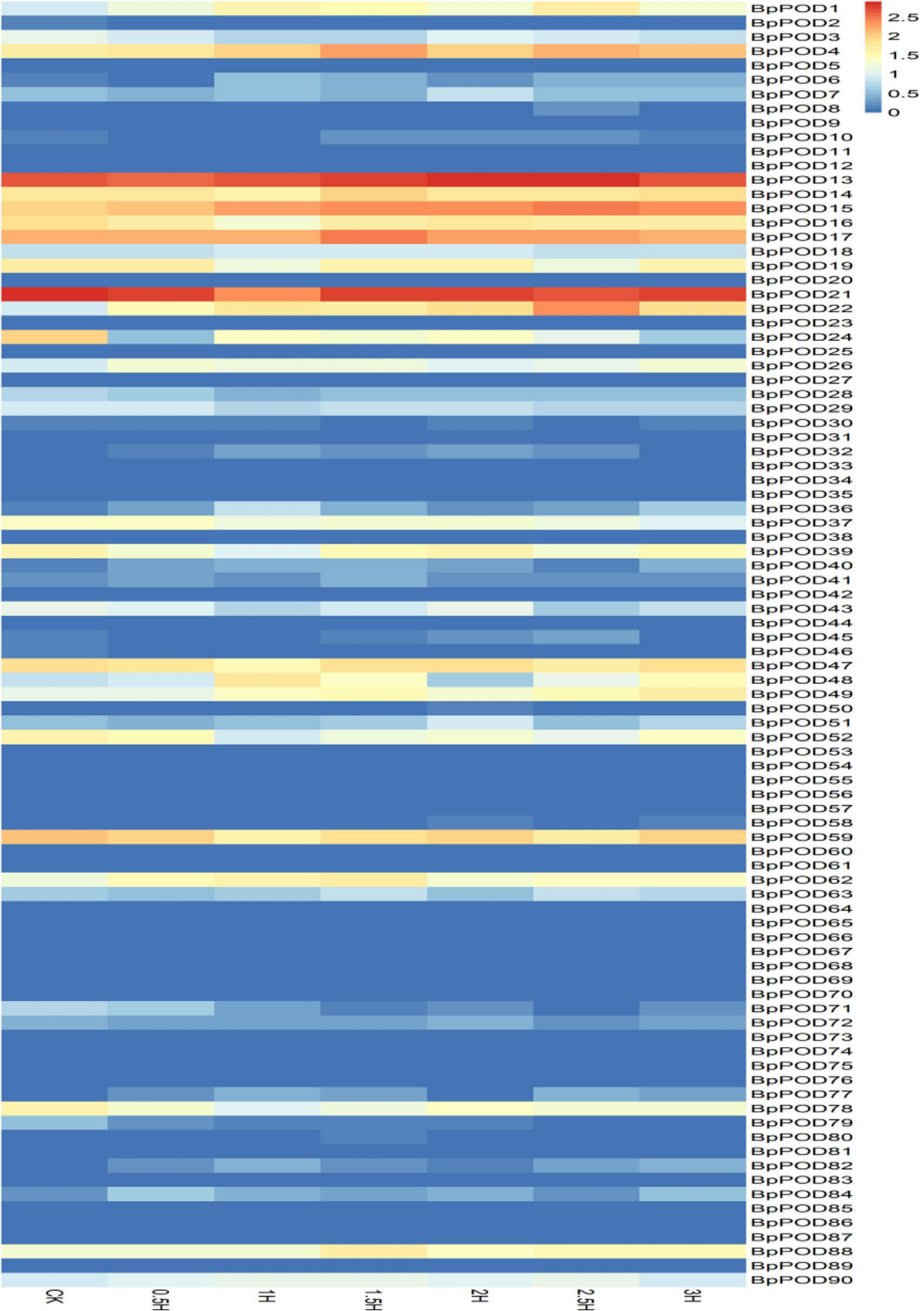
Responses of *BpPOD* genes expression to cold treatment. Different time are exhibited below each column. The *BpPOD* genes were listed at the right of the expression array, and the colour box from blue (0) to orange (2.5) indicate an increased expression level is shown at the right of the figure. Color scale represent the normalized value of the expression. For the sake of unified comparison, the normalized value of the expression was log10 transformed

POD participates in a variety of physiological processes in the plant, and especially in resisting various stresses play an important role [42]. In recent years, many scholars have investigated the performance of *POD* genes in response to abiotic stress [36]. For example, *Arabidopsis* overexpressing *AtPOD3* showed an increase in dehydration and salt tolerance, whereas the antisense suppression of *AtPOD3* exhibited dehydration and salt sensitive phenotypes [43]. In this study, we examined the expression levels of the *BpPODs* in response to low temperature stress. As shown in Fig. 8, the result indicated that the expression of *BpPODs* was altered under cold treatment, some of *BpPODs* are induced but most of them not or slightly induced. After cold treatment, the expression levels of *BpPOD4, BpPOD13, BpPOD15, BpPOD17* and *BpPOD21* were significantly induced at a relatively early stage (0.5 h after treatment), and with the increase of cold treatment time, the relative expression level of these genes was also at a high level. The Fig. 8 shows that the expression levels of *BpPOD19*, *BpPOD21, BpPOD39* and *BpPOD47* were increased after 1.5 h treatment of low temperature. *BpPOD50* and *BpPOD58* did not respond to cold treatment at the beginning (0.5 h), and were slightly increased after 2 h exposure to low temperature. In addition, other genes are also induced by cold stress, such as *BpPOD14*, *BpPOD16*, *BpPOD59*, etc. In general, the *BpPODs* may play important roles in birch under cold stress.

### Validation of transcriptome data by qRT-qPCR analysis

**Fig. 9:**
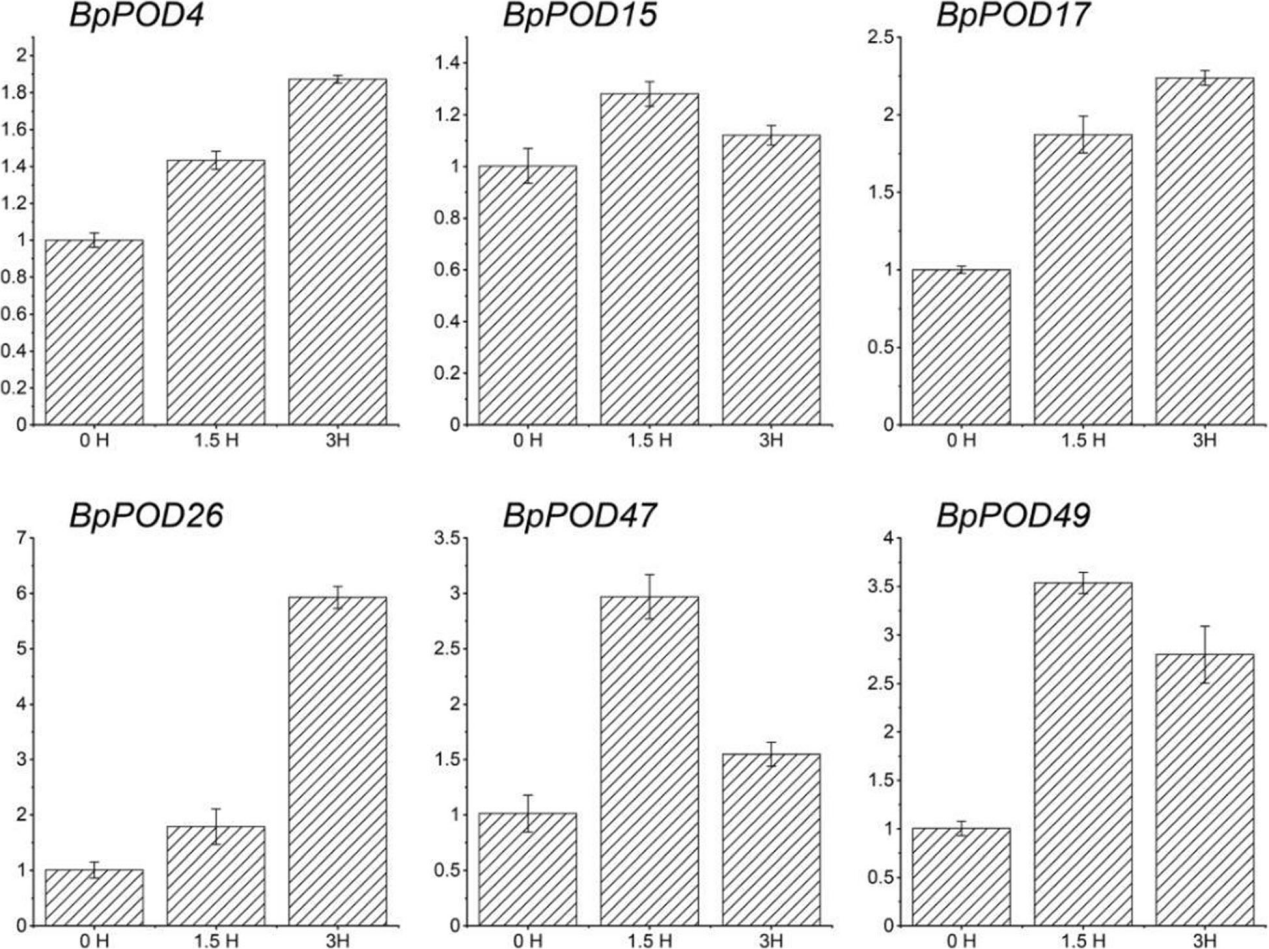
qRT-PCR analysis of *POD* genes in *B. pendula* under cold stress. The X-axis represents the time course of stress treatment and the Y-axis represents the relative expression level. Seedlings were sampled at 0, 1.5 and 3 after cold treatment. Data represent means ± SD in three replicates

To verify the accuracy of the RNA-Seq data under cold treatment (6 °C) in *B. pendula*, six randomly selected *BpPODs* were tested for Quantitative real-time PCR (qRT-PCR). The expression pattern of six *BpPODs* using qRT-qPCR were in accordance with that detected by RNA-seq (Fig. 9). *BpPODs* including *BpPOD15*, *BpPOD47* and *BpPOD49* showed the highest transcript level when exposed to a low temperature for 1.5 h. *BpPOD4*, *BpPOD17* and *BpPOD26* showed the highest transcript level at 3 h. In general, all the results indicated that the expression profile results of RNA-seq were reliable.

## Discussion

It is reported that Class III Peroxidases participates in a variety of physiological processes in the plant [6, 34], and play a important role in biological and abiotic stress responses during plant development [36]. At present, *POD* gene family have been published for *Arabidopsis thaliana* [6], *Populus trichocarpa* [34], *Zea mays* [36] and *Oryza sativa* [11], but there are no reports on the identification and function of *POD* gene family in *Betula pendula.* Fortunately, with the completion of the complete genome sequence of *B. pendula* [29, 30], bioinformatics analysis of the *POD* gene family in *B. pendula* at the genome level has become possible.

In the present study, based on the genomic information of *B. pendula*, a total of 90 *POD* gene family members were identified, the number of *POD* family members was higher than that of *Arabidopsis* (73), which was similar to that of *Populus trichocarpa* (93) and *Pyrus bretschneideri* (94). Subsequently, phylogenetic relationships, subcellular localization, conserved motifs, gene structure and other information were analyzed [44].

In the process of genome evolution, gene duplication was the main factors that led to the expansion of gene family [38]. It has been reported that tandem duplication plays an important role in gene family extension in *B. pendula* [36]. For example, Chen, et al. found that tandem duplication is the main reason for the expansion of the NAC gene family in *B. pendula* [16]. However, in pears, segmental duplication is the main driver of gene family expansion [31]. Interestingly, in this study, we found that some *BpPODs* were adjacent to each other, suggesting tandem duplications play the major role in the evolution of the *BpPOD* gene family. Noteworthy, segmental and tandem duplication contributed to the evolution of *POD* gene family in maize [36]. The results may be one of the reasons why the number of *POD* genes varies among different species. In addition, Ka/Ks analysis showed that the Ka/Ks value of most *BpPOD* gene pairs were less than 1, indicating that these genes underwent negative selection. Furthermore, *BpPOD5/− 6*, *BpPOD24/− 25* and *BpPOD24/− 27* gene pairs had higher Ka/Ks values than other gene pairs, indicating that these genes evolved rapidly and had relatively stable structures. We also constructed the comparative syntenic maps of birch associated with *Arabidopsis thaliana, Populus trichocarpa* and *Vitis vinifera*. The results showed that there are 49 pairs of orthologous gene between birch and *Populus trichocarpa*, while the number of orthologous gene pairs (17) between *Arabidopsis* and birch is relatively small, which may be due to the genetic relationship between *Populus trichocarpa* and birch is close, but the relationship with *Arabidopsis* is far away.

In the study, the 90 BpPOD proteins possess ten highly conserved motifs. In addition, we found that the number and type of conserved motifs in 90 BpPOD proteins were slightly different. Notably, most BpPOD proteins contain all the conserved motifs, while only a few BpPOD proteins contain one or two motifs, which means that these motifs may be involved in the important basic function of the POD protein. The diversity of gene structure plays an important role in the evolution of gene families [45, 46]. In this study, we performed the structure of *BpPOD* genes. The results showed that 90 *BpPOD* genes contained different number of exons and introns, and the characteristics of *BpPODs* from different subgroup were different. These results indicated that the POD gene family of *B. pendula* has great diversity. In addition, the study of gene structure also found that some *BpPODs* lack introns, which may be caused by a specific pathway [10, 47].

RNA-seq is usually used to study the mRNA expression amount of specific tissue or cells transcribed during a certain period of time, and then to analyze the related genes and phenotypes [48]. In this study, we used the acquired transcriptome data to investigate the function of *BpPODs*. RNA-Seq analysis of different tissues found that different *BpPODs* have tissue expression specificity, indicating that *BpPODs* had diverse functions. We found that of the 90 *BpPODs*, the most abundant expression was in the root, followed by the xylem. The results showed that most of the expressed POD genes participated in the reproductive growth process. The highest expression levels of *BpPOD6, BpPOD21* and *BpPOD37* genes were found in xylem. The results implied that three genes may play an important role of xylem synthesis in *B. pendula. BpPOD59* is most expressed in flowers and leaves, suggesting that it may be related to leaf spreading and flowering formation in *B. pendula.* In addition, some *BpPODs* were expressed in all tissues, implying that they may have important effects on the growth and development process in *B. pendula.* In conclusion, *POD* family genes play an important regulatory role in the growth and development of *B. pendula.*

Abiotic stresses such as drought, low temperature and high salinity are serious natural disasters in plants, which seriously affect the growth and development of plants [46]. Plants have established a series of signal transduction and regulation molecular mechanisms to improve their ability to cope with adversity stress [49, 50]. A large number of experimental studies [36] on stress treatment showed that under the stress of low temperature and other conditions, *POD* genes expression increased significantly [25, 26]. However, there are few studies on the response of *POD* genes to cold stress in *B. pendula.* Therefore, we studied the expression patterns of the *BpPODs* under cold treatment. The results suggested that some of *BpPODs* are induced but most of them not or slightly induced. A small number of *BpPODs* were highly expressed at 0.5 h after treatment, and with the extension of time, the expression reached the highest level. This suggested that these genes may be important in the process of resistance to stress in *B. pendula.* By contrast, the expression level of *BpPOD30* and *BpPOD8* gradually increased at 2 h after treatment, implying that these genes participated in the late reaction of cold treatment. In addition, the expression of a few *BpPODs* decreased under cold treatment, we speculate that these genes may also have defense and other specific functions in *B. pendula.* These results suggested that *BpPOD* genes play an important regulatory role in the stress response.

## Conclusion

In short, we identified 90 *POD* genes in *Betula pendula*. According to phylogenetic relationships, these *POD* genes were classified into 12 groups. The *BpPODs* are distributed in different numbers on the 14 chromosomes. In addition, we identified eight conserved domains of BpPOD proteins. Finally, expression patterns analysis revealed that some *BpPODs* might play significant roles in root, xylem, leaf and flower. Furthermore, under low temperature conditions, some *BpPODs* showed different expression patterns at different times. In this study, a preliminary study was conducted on the *POD* genes in *B. pendula*, which laid a foundation for further research on the function of *POD* gene family in future.

## Methods

### Identification of peroxidase genes in *B. pendula*

To identify *B. pendula* peroxidase genes, the *B. pendula* genome sequences were downloaded from National Center for Biotechnology Information ((https://genomevolution.org/CoGe/GenomeInfo.pl?gid=35079)). We also downloaded all annotated POD proteins sequences of *Arabidopsis* from the TAIR database (http://www.arabidopsis.org/). The POD family protein sequence of *Arabidopsis thaliana* was used as seed sequence, and the whole genome of *B. pendula* was searched by BLASTP. To verify the reliability of the results, all the acquired candidate sequences were examined for the presence of the POD domain using PFAM [51] and SMART [52]. Finally, all candidate *POD* sequences were compared by ClustalW [53] and redundant genes were manually checked and removed, and all non-redundant *POD* genes were used for further analysis. The theoretical molecular weights (MWs) and isoelectric points (pIs) of the BpPOD protein sequences were analyzed by the ExPASY PROTPARAM tools (http://web.expasy.org/protparam/) [54].

### Phylogenetic analyses of peroxidase genes in *B. pendula*

To investigate the phylogenetic information of the peroxidase genes of *B. pendula*, an unrooted tree was constructed using amino acid sequences of the peroxidase genes. The MUSCLE with default parameters were used for multi-sequence alignment analysis [55]. Subsequently, The phylogenetic tree was constructed by using MEGA 7.0 software, which was constructed by neighbor-joining method and repeated 1000 times (Bootstrap: 1000). The phylogenetic tree was beautified and annotated by using the online tool ITOL (https://itol.embl.de/).

### Gene structure and conserved motif analysis

The CDS sequences of *PODs* were extracted from the genomic structure information (GFF) of the genome (https://genomevolution.org/CoGe/GenomeInfo.pl?gid=35079), and the intron and exon structures were visually analyzed using Gene Structure Display Server [56]. MEME software was used to analyze the conserved motif of BpPOD proteins [57], and TBtools was used to draw the schematic diagram.

### Chromosomal localization and gene collinearity analysis

According to *BpPODs* starting positions on the birch chromosomes, TBtools software was used to determine the chromosome location image of the *BpPODs* [58]. In addition, the rate of Ka/Ks was calculated for the duplicated gene pairs by using TBtools [58]. For gene collinearity analysis, syntenic maps of birch associated with three representative species were visualized by MCScanX [59].

### Differential expression profile of *BpPOD* gene family

To determine the expression patterns of *BpPODs* in different tissues in *Betula platyphylla* × *Betula pendula*, we downloaded the sequencing data from the NCBI SRA database with an accession number of PRJNA535361 (https://www.ncbi.nlm.nih.gov/sra/?term=PRJNA535361) [16]. To identify the expression of *BpPODs* during cold treatment in *Betula platyphylla* × *Betula pendula*, we designed the experiment including six time points. In this study, two-month-old *Betula platyphylla × Betula pendula* plants grown in the greenhouse of Northeast Forestry University were exposed to low temperatures (6 °C) for 0.5 h, 1 h, 1.5 h, 2 h, 2.5 h, and 3 h, respectively [16]. In addition, plants without cold treatment were used as the control. After cold treatment, all young leaves were harvested at the same time to avoid changes in gene expression due to different harvest times. Total RNA samples were isolated from the leaves using the RNAprep Kit. The constructed cDNA libraries were sequenced using the Illumina HiSeq platform at Biomarker Technologies Corporation (Beijing, China). We can downloaded the sequencing data from the NCBI SRA database with an accession number of PRJNA532995 (https://www.ncbi.nlm.nih.gov/sra/?term=PRJNA532995) [16].

### qRT-PCR test

To evaluate the reliability of the RNA-seq data, six randomly *BpPODs* with cold treatment were selected and examined by qRT-PCR analysis. Total RNA of leaves of collected samples were extracted and purified using DNase I digestion (Takara, Dalian, China) to remove mixed DNA. Quantitative real-time RT-PCR was performed on an ABI 7500 Real-Time system (Applied Biosystems). The primers were designed using A plasmid Editor v1.11 (Table 6), and 18S rRNA was used as a reference gene. The PCR reaction protocol was conducted with 20 μl volume containing 94 °C for 30s, followed by 45 cycles of 94 °C for 5 s, 60 °C for 35 s, 95 °C for 15 s, 60 °C for 1 min, followed by 95 °C for 15 s. The relative expression level was determined according to the 2^−ΔΔCT^ method. Three biological replicates were carried out for each sample.

**Table 6:** BpPOD gene-specific primers used for qRT-PCR analysis

ID	BpPOD gene name	Primer sequences (5′ to 3′)
1	BpPOD4-F	GTGGAGTTGGGAAGACTAGATGG
2	BpPOD4-R	GCAATCATATCGGTTTGGGTGAG
3	BpPOD15-F	TCTTGCCTTCTCCCAATTCTACC
4	BpPOD15-R	GAAAACTACACACCGTGCTTCTC
5	BpPOD17-F	CTATCCTCCGCTTGTTTTTCCAC
6	BpPOD17-R	TCTGACAGAGTTTCGATTGGGAG
7	BpPOD26-F	GTGGCCTAATCACTTCCTTCTCA
8	BpPOD26-R	TGTTGGACTAGTGACGTCAAGAG
9	BpPOD47-F	CAAACGTTGAGTCTACTGTGCAG
10	BpPOD47-R	TACAGTGTCAAACCCATCTCCTG
11	BpPOD49-F	TCGGATCAAGCTCTTCTCACAAA
12	BpPOD49-R	AACTACTTTGCAGTCGAGCCTAA
13	18S-F	GAGGTAGCTTCGGGCGCAACT
14	18S-R	GCAGGTTAGCGAAATGCGATAC

## Data Availability

All data generated or analysed during this study are included in this published article.

## Abbreviations

POD: Class III peroxidases
*B. pendula*: *Betula pendula*
*BpPODs*: *POD* genes in *Betula pendula*
RNA-seq: RNA sequencing
